# Vitamin C—Sources, Physiological Role, Kinetics, Deficiency, Use, Toxicity, and Determination

**DOI:** 10.3390/nu13020615

**Published:** 2021-02-13

**Authors:** Martin Doseděl, Eduard Jirkovský, Kateřina Macáková, Lenka Kujovská Krčmová, Lenka Javorská, Jana Pourová, Laura Mercolini, Fernando Remião, Lucie Nováková, Přemysl Mladěnka, on behalf of The OEMONOM

**Affiliations:** 1 Department of Social and Clinical Pharmacy, Faculty of Pharmacy, Charles University, 500 05 Hradec Králové, Czech Republic; martin.dosedel@faf.cuni.cz; 2 Department of Pharmacology and Toxicology, Faculty of Pharmacy, Charles University, 500 05 Hradec Králové, Czech Republic; JirkovskyE@faf.cuni.cz (E.J.); pourova@faf.cuni.cz (J.P.); 3 Department of Pharmacognosy, Faculty of Pharmacy, Charles University, 500 05 Hradec Králové, Czech Republic; macakovak@faf.cuni.cz; 4 Department of Analytical Chemistry, Faculty of Pharmacy, Charles University, 500 05 Hradec Králové, Czech Republic; KRCML1AA@faf.cuni.cz (L.K.K.); novakoval@faf.cuni.cz (L.N.); 5 Department of Clinical Biochemistry and Diagnostics, University Hospital Hradec Králové, 500 05 Hradec Králové, Czech Republic; lenka.javorska@fnhk.cz; 6 Research group of Pharmaco-Toxicological Analysis (PTA Lab), Department of Pharmacy and Biotechnology (FaBiT), Alma Mater Studiorum—University of Bologna, 40126 Bologna, Italy; laura.mercolini@unibo.it; 7 UCIBIO-REQUIMTE, Laboratory of Toxicology, Biological Sciences Department, Faculty of Pharmacy, University of Porto, 4050-313 Porto, Portugal; remiao@ff.up.pt

**Keywords:** ascorbic acid, antioxidant, prooxidant, scurvy, oxalate, epigenetic

## Abstract

Vitamin C (L-ascorbic acid) has been known as an antioxidant for most people. However, its physiological role is much larger and encompasses very different processes ranging from facilitation of iron absorption through involvement in hormones and carnitine synthesis for important roles in epigenetic processes. Contrarily, high doses act as a pro-oxidant than an anti-oxidant. This may also be the reason why plasma levels are meticulously regulated on the level of absorption and excretion in the kidney. Interestingly, most cells contain vitamin C in millimolar concentrations, which is much higher than its plasma concentrations, and compared to other vitamins. The role of vitamin C is well demonstrated by miscellaneous symptoms of its absence—scurvy. The only clinically well-documented indication for vitamin C is scurvy. The effects of vitamin C administration on cancer, cardiovascular diseases, and infections are rather minor or even debatable in the general population. Vitamin C is relatively safe, but caution should be given to the administration of high doses, which can cause overt side effects in some susceptible patients (e.g., oxalate renal stones). Lastly, analytical methods for its determination with advantages and pitfalls are also discussed in this review.

## 1. Introduction

Vitamin C is known as L-ascorbic acid since it was observed as the factor needed for the treatment of scurvy (in Latin *scorbutus*, hence, “a-scorbutus”). Since the terms “fat-soluble vitamin A” and “water-soluble vitamin B” were already in use, the term vitamin C was coined. In general, the term also includes its oxidized form, L-dehydroascorbic acid, which can easily be converted into L-ascorbic acid in the human body.

In contrast to most of the vertebrates, guinea pigs, bats, passeriform birds, and primates, including humans, lack L-gulono-1,4-lactone oxidase, and, hence, cannot synthesize vitamin C. They are, therefore, fully dependent on vitamin C intake from the diet [1,2,3]. Vitamin C is very popular among the general population mainly due to its antioxidant properties. Its role is, however, more extensive and will be discussed in this review. This is reflected by the body content of vitamin C that is unusually high compared to other vitamins. The human body was suggested to contain about 1.5 g of vitamin C, which corresponds to around 20 mg/kg. The daily need in humans is hardly or at all assessable since it depends on many factors, including physiological status, stress, and diseases. Recommendations differ substantially from 40 to 120 mg/day in various countries. This dose represents the assessment of the normal need for vitamin C in humans. In pregnant women, an additional 5–10 mg is recommended (25 mg in lactating mothers) [3,4,5,6]. The total daily dose of about 10 mg is sufficient to prevent scurvy in the general population [3].

**Figure 1:**
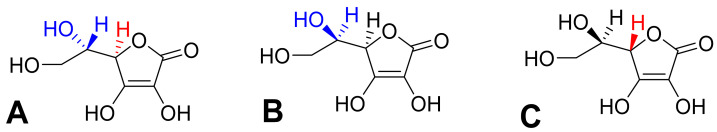
Optical isomers of vitamin C. (**A**) L-ascorbic acid, (**B**) erythorbic acid, and (**C**) D-ascorbic acid. Differences are shown in red and blue.

This review is devoted to the biological aspects of L-ascorbic acid (Figure 1A). Its epimer erythorbic acid (Figure 1B, also known as isoascorbic acid or D-araboascorbic acid), which is used as a food additive, and its enantiomer D-ascorbic acid (Figure 1C) are also partly active as vitamin C. However, they are only poorly retained by tissues and are more rapidly excreted from the human body [7,8,9,10]. 

## 2. Sources

In contrast to many other vitamins, the content of vitamin C in various foods is, in general, relatively high (10–100 mg/100 g), and, in some cases, it reaches units of grams per 100 g of fresh weight. This is possibly related to the fact that vitamin C is formed from sugars, which are common compounds in different organisms. So far, the synthesis of vitamin C was documented in all plant species, including algae and photosynthetic protists [1]. In plants, L-ascorbic acid is responsible for three main functions: an enzyme cofactor, a radical scavenger, and a donor/acceptor in electron transport either in the plasma membrane or in the chloroplasts, besides other minor functions [11]. Currently, most of the daily intake of vitamin C comes from fruits and vegetables, which, in many countries, unlike in the past, are available throughout the year. A large part is also obtained from potatoes and soft drinks, including juices. Although most vertebrates are able to synthesize vitamin C, animal sources do not contribute much to the intake because the content of vitamin C in them is generally low, except for livestock livers, which are consumed minimally, and some fish eggs. However, they are of the utmost importance to nations living in subarctic areas [12,13]. Besides plants and animals, fungi are also capable of biosynthesis of vitamin C. However, its content in both wild and cultivated fungi is generally very low [14,15,16].

Several fruits from different parts of the world, including kakadu plum from Australia, camu-camu, and acerola from South America, are the richest vitamin C sources (Table 1) [17,18,19]. In Europe and Asia, rose hips and sea buckthorn are considered the richest sources of this vitamin [20,21]. Most people get a large portion of their daily vitamin C intake through regular consumption of fruits and fruit juices. The species composition varies by region. Globally, star fruit, guava, black currant, kiwi, and strawberry are good sources. Compared to the richest sources of the vitamin, citruses contain a significantly lower but sufficient amount of vitamin C [22,23,24,25]. Considering vegetables, cruciferous vegetables, especially broccoli, kale, and peppers, are also rich sources [26,27]. The content of vitamin C in fermented cabbage (sauerkraut) is even higher than in most fresh vegetables [28]. In contrast, potatoes have a relatively low vitamin C content but play an important role in its intake because they are consumed in large quantities [29]. Notwithstanding the fact that they are consumed boiled, they are a suitable source of vitamin C. A single serving of potatoes offers 5–40 mg of vitamin C. In recent years, they have accounted for an average of 8% of the total daily intake of vitamin C in European countries [3,12]. Fresh aromatic herbs (coriander, parsley, chives), which are a frequent part of vegetable salads, also make a significant contribution to their overall vitamin C content [30]. Algae belonging to different taxonomic groups are also not negligible sources. Their vitamin C content is in the tens of mg by 100 g [31]. As can be seen from Table 1, the vitamin C content of individual plant species is highly variable due to many factors, including place of cultivation, harvesting time (ripening stage), weather conditions, latitude, genotype, agrotechnology, analytical method used for determination, and processing [24,32,33,34,35].

Aqueous vitamin C solutions are unstable since oxygen and other oxidizing agents, high pH, high temperature, and metal ions cause its decomposition. Long-term cooking and, in particular, boiling with a large amount of water results in leaching of vitamin C into the water and, hence, markedly decreases the vitamin C content in food. Even blanching leads to relatively large losses of vitamin C. Surprisingly, frying can retain a sufficient amount of the vitamin. However, steaming or boiling in a small amount of water seem to be more gentle ways in relation to vitamin C preservation. Losses occur even if all external factors are eliminated to a minimum because oxidation reactions take place inside the material due to the presence of ascorbic acid oxidase. Thus, the ideal processing method is the rapid thermal inactivation of the enzyme with a minimal amount of water followed by rapid cooling. The temperature also has a large impact on storage stability. With increasing temperature, more significant losses occur. However, during long-term storage, the amount of vitamin C decreases significantly even if conditions under which only small losses occur during short-term storage are maintained. Post-harvest losses are mainly due to an enzyme-catalyzed oxidation reaction, which degree depends especially on pH, material integrity, and temperature. Summing up, the gentlest way to preserve fruits and vegetables for their content of vitamin C for a longer time is deep-freezing [3,5,11,36,37,38].

Industrially, most of the L-ascorbic acid is currently produced by two fermentation processes requiring several chemically-based steps: the Reichstein process and the two-step fermentation process. The Reichstein process is based on the catalytic hydrogenation of D-glucose to D-sorbitol and subsequent bioconversion to L-sorbose using *Gluconobacter* spp., which is followed by oxidation of L-sorbose to 2-keto-L-gulonic acid. This is rearranged to L-ascorbic acid by lactonization. In the second production process, the chemical production of 2-keto-L-gulonic acid from L-sorbose is replaced by bioconversion using various bacteria [39]. Although the bioavailability of biotechnologically prepared and plant(food)-derived vitamin C seems to be comparable as described in the absorption section, many people prefer vitamin C of natural origin, including both food and food supplements [40]. The richest sources of vitamin C, such as kakadu plum, camu-camu, acerola, rosehips, and sea buckthorn fruits, are mainly used to produce food supplements (Table 1).

## 3. Kinetics of Vitamin C

### 3.1. Absorption

**Figure 2:**
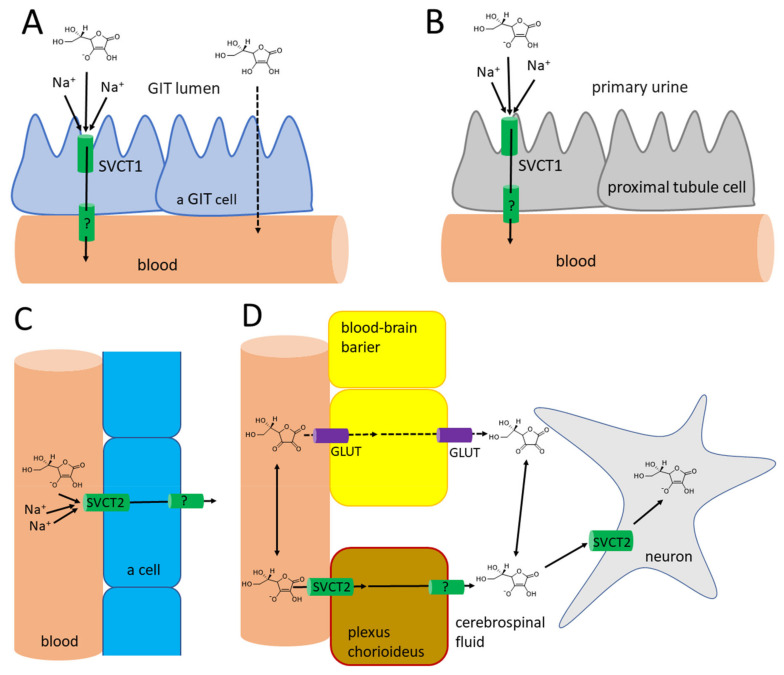
Vitamin C kinetics in the human body. (**A**): Absorption in the gastrointestinal tract. In the distal ileum, the absorption of ascorbate is mediated via SVCT1, while in the upper parts of the GIT, where pH is lower, the passive diffusion of non-ionized ascorbic acid is also possible. Absorption of dehydroascorbic acid does not seem to contribute significantly and is not shown. (**B**): reabsorption of vitamin C through the proximal tubules to the blood. Passive diffusion is also possible in acidic urine, likely in other parts of the urinary tract, but it does not seem to contribute significantly to vitamin C reabsorption and is, hence, not shown. (**C**): Distribution of vitamin C to most cells. (**D**): Distribution of vitamin C to the neurons. There are no specific transporters for ascorbate in the blood-brain barrier. Hence, the only possible way is the uptake and release of vitamin C in the form of dehydroascorbic acid via glucose transporters (GLUT). This transport is likely not the major contributor of vitamin C distribution to the brain (see corresponding part of the article). Contrarily, in the choroid plexus, the SVCT2 is expressed and this seems to be the major pathway for vitamin C kinetics to the brain. Neurons are also expressing SVCT2.

**Figure 3:**
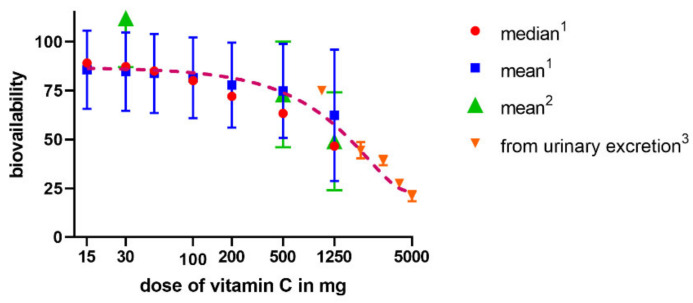
Relationship between a dose of vitamin C and bioavailability in humans. The data are from three studies—^1^ Graumlich et al. 1997 [57], ^2^ Levine et al., 1996 [55], and ^3^ Hornig et al., 1980 [56].

The absorption of vitamin C takes place mostly in the distal ileum. The transport to enterocytes is mediated by SVCT1 (**s**odium-dependent vitamin C transporter 1, solute carrier of the family of ascorbate transporters, SLC23A1, Figure 2A). It is, therefore, saturable and sodium dependent. The Michaelis constant K_m_, i.e., the concentration of vitamin C transported by half of the maximal transport velocity, was reported to be in a wide range from 10 to 200 µM, suggesting a large capacity to absorb dietary vitamin C. Its activity is highest at a neutral pH. It drops markedly with increasing acidity [51,52,53,54]. Some passive diffusion in the gastrointestinal tract (GIT) cannot be fully excluded since vitamin C is present in the non-ionized form at a low pH. This can be supported by the fact that its isomer, erythorbic acid, which is a poor substrate of SVCT1, reaches similar plasma concentrations as vitamin C [52]. Based on the saturable mechanism of vitamin C transport via SVCT1, it is not surprising that the absorption is not linear. The bioavailability decreases with an increasing dose being 100% in a low dose of 200 mg and dropping to an average of 73% at a dose of 500 mg and to approximately 50% in the dose of 1.25 g [55]. Higher doses have even lower bioavailability [56]. Later, a more sophisticated bioavailability calculation suggested slightly different values [57] (see Figure 3). There is a discussion whether synthetic or pure vitamin C has better or lower bioavailability than dietary vitamin C. Some suggestions were coming from experimental studies that diet rich in flavonoids can improve it, but the data are inconclusive [40]. This is likely due to the fact that vitamin C has high bioavailability. Hence, minor changes in this parameter are not easily detectable considering physiological inter-individual variability, which can also be seen in Figure 3.

The oxidized form of vitamin C, dehydroascorbic acid, is absorbed likely through glucose transporters, and glucose decreases absorption of dehydroascorbic acid. In any case, the contribution of dehydroascorbic acid to the total vitamin C absorption is probably low [52]. 

The efflux of ascorbic acid from GIT cells to the circulation is likely mediated by a transporter, but its identity is currently unknown. Dehydroascorbic acid can be again transported through the basolateral membrane of these cells by glucose transporters [52]. In general, vitamin C absorption is relatively slow, as maximal plasma levels are reached after 2–4 h [6,8,40,58].

### 3.2. Distribution and Metabolism

The average plasma levels of vitamin C in a healthy adult population is between 40 and 65 µM. There is no difference between serum and plasma [59,60,61,62,63]. The levels of vitamin C fluctuate with age. They are highest in the category of 6 to 11 years old and then gradually decrease. There is, however, an increase in the population over 60 years old in both men and women [61]. Women have, in general, higher plasma levels than men [61]. The maximal steady-state, long-term vitamin C plasma levels achievable by oral administration are 70–85 µM [5,64]. The plasma levels increase to this plateau concentration up to doses of about 200–400 mg daily. Higher doses increase plasma levels only minimally [55,65,66]. Concentrations up to 220 µM can also be reached for a short time, but this requires a maximum tolerable dose of 3 g every 4 hours [64]. It is well known that smoking affects vitamin C plasma levels negatively [60,61]. Smoking decreases the plasma level of vitamin C on an average by 25–50% [52,61,63]. Ex-smoking seems to slightly decrease the levels as well [52,60]. These lower plasma levels can be at least partly accounted to the increased oxidative stress caused by smoke. The need for vitamin C in smokers is higher, but the available studies suggested different recommendations ranging from 35 to 200 mg of additional vitamin C/day needed for smokers [52]. The plasma levels of dehydro-ascorbic acid are very low in healthy humans, while that of an ascorbyl radical are undetectable [6,53,54,62,67]. Vitamin C is not bound to plasma proteins [62]. 

The pKa of ascorbic acid is 4.1–4.2. Hence, it is present completely as a monoanion—ascorbate at the physiological pH [1,2,4,68]. This form cannot cross the membranes directly. For this reason, transporters are crucial players in the pharmacokinetics of vitamin C. Vitamin C is taken up by the cells via SVCT2 (SLC23A2, Figure 2C), which is a close analogue of SVCT1 with which it shares 65% sequence homology. SVCT2 is largely expressed in most organs. The expression of SVCT1 is much more limited. In addition to the intestine, it is localized in the liver, lung, skin, ovary, prostate, and kidney. In mice, the absence of SVCT2 leads to the absence of the vitamin in the lungs, but the liver is not affected. Like SVCT1, SVCT2-mediated transport is unidirectional and uses an electrochemical sodium gradient. Two sodium ions are needed for the transport of vitamin C by both transporters. SVCT2 has a 2–10 fold higher affinity to vitamin C when compared to SVCT1. However, the V_max_ is apparently lower. This suggests a lower capacity to transport vitamin C, but with higher sensitivity, i.e., the transport is also working in lower concentrations of vitamin C. This agrees with the physiological need for a higher capacity of SVCT1 to transport vitamin C from the diet and the function of SVCT2 to provide vitamin C to the cells even in the presence of low plasma vitamin C [51,52,53,54,69,70]. SVCT2 enables a considerable plasma-tissues concentration gradient. The intracellular levels of vitamin C are much higher than plasma levels. Vitamin C reaches a concentration between 0.5–5 mM in most cells with the exception of erythrocytes, which do not express SVCT2, and, hence, their cytosolic levels reflect plasma levels and are about 50 µM [52,55,68,71]. In contrast to maximal plasma levels reported above, the maximal concentration in different types of white blood cells was achieved by a dose of 100 mg daily. Higher doses in men did not increase this intracellular level substantially [55]. The possible reason can be the saturable kinetics of active transport by SVCT2 with K_m_ of approximately 60–70 µM, which are the approximate plasma levels associated with 100-mg daily dosing [5]. This was not confirmed in women where the saturable doses were 200–400 mg daily, matching with doses producing maximal steady-state plasma levels [66]. Physiologically high levels (2–10 mM) are found in neurons and endocrine cells (particularly, the adrenal and pituitary gland), which is apparently related to the synthesis of hormones and neuro-mediators described below [4,6,8,72,73]. Vitamin C has little tendency to exit the cells likely due its hydrophilic nature and negative charge at a physiological pH [73]. In extracellular fluid, the concentrations are slightly higher than in plasma, but, in general, they reflect plasma levels [67].

**Figure 4:**
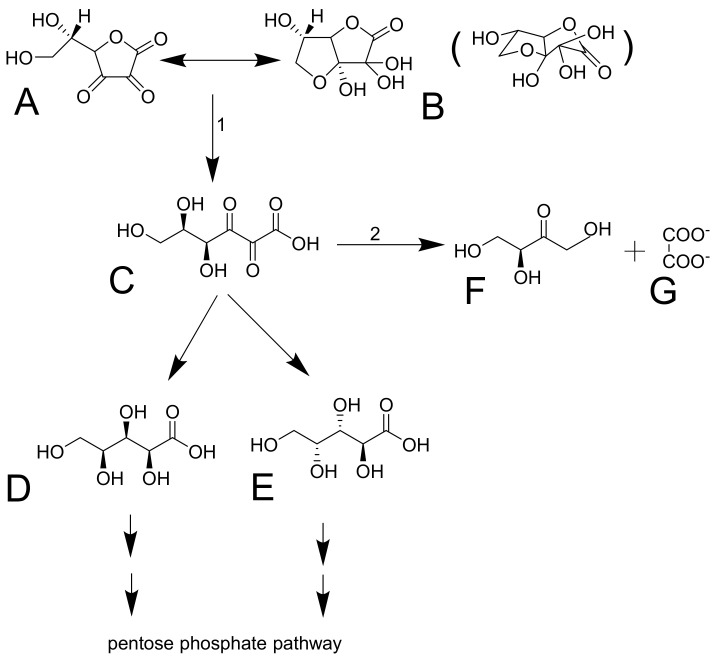
Dehydroascorbic acid and its decomposition. Dehydroascorbic acid (**A**) forms reversibly a hemiketal (**B**, a spatial structure shown in brackets). The structure is not stable and it is irreversibly transformed in 2,3-diketo-1-gulonic acid (**C**). This compound can be decarboxylated into L-xylonic acid (**D**) or L-lyxonic acid (**E**) or in L-erythrulose (**F**) and oxalate (**G**). 1: the reaction can be both spontaneous or mediated by an enzyme. 2: No enzyme mediating this reaction was reported. The reaction is likely spontaneous.

Dehydroascorbic acid does not resemble glucose, but it forms bicyclic hemiketal (Figure 4A,B), which resembles glucose [74,75] and has high affinity for glucose transporters GLUT1 and GLUT3 (GLUT4 can be involved, but GLUT2 and GLUT5 are not) [54]. These transporters mediate facilitated diffusion and are, therefore, bidirectional. Since the concentration of dehydroascorbic acid in plasma is low, the oxidized form of vitamin C in the cells is likely rapidly reduced to ascorbic acid. This seems to be typical for erythrocytes. The reduction is the crucial step since dehydroascorbic acid is not very stable. It has a half-life of about 6 minutes. It is decomposed to 2,3-diketo-1-gulonic acid (Figure 4) and, in this way, it loses its activity of vitamin C [6,68,73]. There are apparently redundant pathways mediating the reduction of dehydroascorbic acid to ascorbic acid. They include both direct but slow nonenzymatic reduction by glutathione and enzymatic by a couple of enzymes (e.g., glutaredoxin, glutathione transferase omega 2, or thioredoxin reductase). Of note is that the first two enzymes require glutathione for their activity [1,73]. Erythrocytes are very active in mediating this reduction [6,52]. After the reduction to 2,3-diketo-1-gulonic acid, several scenarios may follow. It can be decarboxylated (Figure 4D,E) and the products can enter the pentose phosphate shunt by a couple of reactions [76]. 2,3-diketo-1-gulonic acid can be spontaneously, but also enzymatically, decomposed to erythrulose and oxalate. This reaction is accelerated by bicarbonate [1,3].

An interesting aspect is the transport of vitamin C to the central nervous system (Figure 2D). The current knowledge suggests that vitamin C is transported to cerebrospinal fluid in the choroid plexus via SVCT2. There are also reports that vitamin C can pass through the blood-brain barrier in the form of dehydroascorbic acid through the glucose transporter GLUT1 [52,53,73]. Although experimental data reported that penetration of dehydroascorbic acid is apparently much more intense and rapid than that of ascorbic acid [77], physiologically, this transport has likely minor importance. Dehydroascorbic acid is unstable and has very low plasma levels, so its competition with physiological low millimolar plasma levels of glucose for these transporters is rendering that mechanism of uptake of vitamin C in this form via GLUT1 disputable [53,68]. Moreover, the absence of SVCT2 in rodents results in very low levels of vitamin C in the brain, clearly emphasizing the importance of this transport for the brain [69,70]. The uptake of vitamin C by neuronal cells is very intense and is mediated mainly by SVCT2. GLUT1/3 can also contribute to transporting dehydroascorbic acid to neuronal cells. Neurons possess very high intracellular concentrations of vitamin C, which can reach 10 mM, while the concentration in glial cells is similar to other body cells. The cerebrospinal fluid levels are about 200 µM, which is about four times higher than the average plasma levels. Vitamin C is apparently crucial for the correct functions of the brain and its levels in central nervous system are less affected by vitamin C starving than other tissues [73]. The absence of SVCT2 is incompatible with life [69,70]. It was shown that the expression of SVCT2 is increased after experimental vascular brain injury [53].

According to animal studies, SVCT2 is also important for the transport of vitamin C from the mother to the fetus, but SVCT1 likely participates in this process [69,70,78]. The plasma levels of vitamin C in the fetus are higher than in the mother in early pregnancy, which apparently suggests that the fetal uptake of vitamin C is preferred at the expense of the mother [78].

### 3.3. Excretion

Radioactive labelled vitamin C administered to three humans showed that most of the amount is eliminated by the kidney. Only less than 1% was eliminated by the feces and virtually no vitamin C was breathed out as carbon dioxide. About 20% of urinary excretion accounted for unmetabolized ascorbic acid, the other 20% for 2,3-diketo-1-gulonic acid, only 2% for dehydroascorbic acid, while, on average, 44% was eliminated in the form of oxalate [79]. Vitamin C is efficiently and saturably reabsorbed from the urinary tract, and SVCT1 plays an important role, as documented by knockout Slc23a1^−/−^ mice with an 18-fold higher ascorbate excretion [51,80,81]. This means that the excess of vitamin C is efficiently eliminated in the urine to maintain the homeostatic vitamin C plasma levels. The player responsible for the saturable reabsorption is, like in the intestine, the SVCT1 transporter, which is expressed on the brush border of the proximal tubules (Figure 2B). Since the pH of urine is also lower, passive diffusion was suggested but apparently plays a minor, if any, role at all [52,53]. The saturable mechanism of reabsorption is responsible for the maintenance of vitamin C plasma concentration together with the saturability of absorption. In daily doses of 30–60 mg, almost no vitamin C is excreted in urine within 24 h, while a dose of 100 mg results in 25% of the dose of vitamin C excreted and doses equalling or above 500 mg are almost entirely excreted [55,57]. The Michaelis constant K_m_ for renal reabsorption of vitamin C in relation to its plasma levels was suggested to be 33 µM, which is approximately in line with the maximum plasma levels [57]. The elimination half-life of vitamin C is generally about 2 h [52].

### 3.4. Genetic Polymorphism

The influence of genetic variations on vitamin C pharmacokinetics is based on single nucleotide polymorphisms (SNPs) found in *SLC23A1* and *SLC23A2* genes with a possible association to human diseases (reviewed in References [2,6]). The strongest evidence to decrease circulating vitamin C levels was found for several common SNPs of *SLC23A1* due to reduced renal reabsorption. However, an elevation of vitamin C levels was also seldomly described [80,82,83]. These SNPs are more common in the African and African-American populations over others, while SNPs of *Slc23A2* are of a similar frequency in the Caucasian and African populations [80,82,84]. SNPs of *SLC23A1* were characterized as both synonymous and non-synonymous SNPs (with the greatest diversity found in the African population) producing proteins of lower functionality. In the case of SNPs of *SLC23A2*, all studied polymorphisms were synonymous [80,83]. An effect of individual SNPs on the vitamin C system level has an additive character, which depends on genotype and an allele dosage (additive) effect that was also described. Furthermore, there have also been hundreds of less common or rare SNPs of both genes. Their frequency in populations is unknown, and, therefore, the global significance of SNPs on circulating vitamin C levels as well as their association with diseases needs further investigation [2,83,85].

Some common *SLC23A1* polymorphisms were found to be associated with an increased risk of Crohn’s disease [86], non-Hodgkin lymphoma [87], preterm delivery [84], and aggressive periodontitis [88]. In addition, no associations were reported between one common variation of *SLC23A1* and arterial blood pressure or different metabolic parameters [89]. Common variations of *SLC23A2* were linked to increased or decreased risk of gastric cancer depending on a discrete genotype [85,90], decreased risk of colorectal adenocarcinoma [91], HPV16-positive head and neck cancer [92], increased risk of bladder cancer [93], non-Hodgkin lymphoma [87], chronic lymphocytic leukemia [94], preterm delivery [84], open-angle glaucoma [95], and acute coronary syndrome in women [96].

### 3.5. Physiological Function

**Figure 5:**
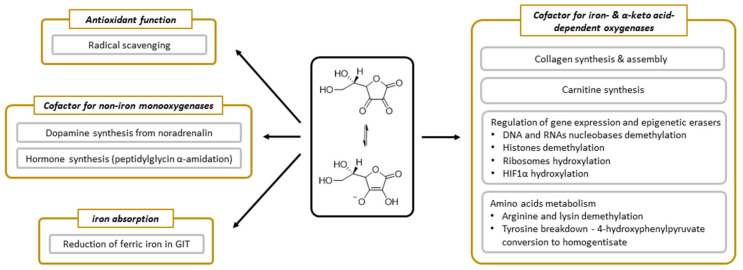
The summary of physiological functions of vitamin C.

Vitamin C has been undergoing extensive research and we know many processes in which it is involved. Its function appears to be linked dominantly with its electron-donating property [6]. Vitamin C participates in several processes that are related to collagen synthesis, synthesis of hormones (noradrenaline/adrenaline and peptide hormones), synthesis of carnitine, gene transcription, and regulation of translation via different mechanisms (hydroxylation of transcription factors, tRNA and ribosomal proteins, demethylation of DNA, and histones), elimination of tyrosine, protection against reactive oxygen species (ROS), and reduction of iron in the gastrointestinal tract (Figure 5).

### 3.6. Vitamin C as an Enzymatic Cofactor

The enzymatic roles of vitamin C are linked with either dioxygenases (synthesis of collagen and carnitine, involvement in gene transcription, and regulation of translation via different mechanisms and elimination of tyrosine) or monooxygenases (synthesis of hormones). All these vitamin C-dependent oxygenases have a metal, iron, or copper in their active site. The involvement of vitamin C in these enzymatic reactions is well documented. The precise mechanism is, however, not fully elucidated, but appears to be related to reduction or maintenance of these metals in the reduced state. In some cases, vitamin C is reduced stoichiometrically within the reaction, suggesting direct involvement. However, in others, the stoichiometry is more complicated, implicating rather that vitamin C can recover the enzymatic function if the central metal atom is oxidized. It should be mentioned that vitamin C seems to be an ideal cofactor for these enzymes. It can be potentially, but not always, replaced by other reductants, which are, however, apparently less active. Individual enzymes and their groups will now be briefly discussed.

### 3.7. 2-Oxoglutarate-Dependent Dioxygenases

The largest group of enzymes using vitamin C as one of their cofactors is the iron-dependent and 2-oxoglutarate-dependent dioxygenases superfamily (2OGO, α-ketoglutarate-dependent hydroxylases). In humans, it represents about 80 enzymes responsible for the modification of important biological substances and processes (Table 2) [97,98,99]. 

The 2OGO were initially identified in the 1970s when studying collagen biosynthesis—a formation of hydroxyproline by collagen prolyl-4-hydroxylase enabling collagen cross-linking and, hence, correct formation of connective tissue. It does not need to be emphasized that vitamin C is essential to avoid the symptoms of scurvy related to impaired formation of connective tissue. The role(s) of vitamin C in this reaction cycle is (are) not fully elucidated, but likely the most important role is to maintain the iron atom in the reduced form [100,101]. In any case, vitamin C seems to be irreplaceable for the physiological function of these enzymes. It seems to be oxidized during the process, but the oxidation is not stoichiometric in relation to the reaction. This can support the theory of maintaining the central atom in its ferrous form and/or cause its reduction in the enzymatic process, in particular when the enzymatic reaction becomes uncoupled [4,68,97,101,102,103,104,105]. A simple, non-selective reduction is rather improbable since glutathione, L-cysteine, or dithiothreitol are inactive. Moreover, the intracellular concentration of vitamin C should be apparently relatively high for the optimal function of these enzymes since K_m_ for vitamin C ranges from 140 to 300 µM [103]. There are also some reports claiming that vitamin C is not needed, but they were questioned since ferrous ions could, at least, briefly substitute the lack of vitamin C [102]. It is possible that there are differences between individual 2OGO as carnitine synthesis, which does not require vitamin C, according to a complex animal study, and the authors suggested that glutathione can replace it in this case [106].

A relatively new discovery is the finding that vitamin C is the specific cofactor of 2OGO involved in cellular stress-signalling and epigenetics (reviewed in References [98,101,102,107,108,109]). It comprises hydroxylases involved in the regulation of the classical cellular sensor hypoxia-inducible factor 1α (HIF1 α), e.g., prolyl-hydroxylase domain-containing proteins (PHDs) and factor inhibiting HIF (FIH) [110,111], and various enzymes involved in epigenetic machinery in humans as epigenetic modification erasers. One of the most known enzyme groups are Jumonji-C (JmjC) domain-containing proteins responsible for hydroxylation of specific histone lysines leading to histone demethylation (JmjC demethylases, e.g., JHDMs and KDMs families) [107,112,113,114]. Ribosomal oxygenases, other members of JmjC domain-containing enzymes with a specific structure containing C-terminal winged-helix (WH)-domains, catalyze histidine-hydroxylation in ribosomal proteins rpL27a (MYC-induced nuclear antigen 53, Mina53), and rpL8 (Nucleolar protein 66, NO66) [115,116]. Another 2OGO involved in epigenetic modifications are ten-eleven translocases (TETs) responsible for hydroxylation of 5-methylcytosine to 5-hydroxymethylcytosine primary in DNA [103,114,117,118,119,120], and RNA and DNA demethylases from the AlkB family. Currently, we distinguish nine AlkB homologues. ALKBH1-ALKBH8 and ALKBH9 (known previously as FTO (Fat mass and obesity-associated protein)), that are responsible for hydroxylation of methylated nucleoside bases with various substrate specificity [121,122,123,124,125]. Of high importance is the high specificity of FTO and ALKBH5 to demethylate the most frequent internal modification of RNAs, N^6^-methyladenine (m^6^A) [126]. Generally, 2OGO catalyze specific hydroxylation of substrates, which, in turn, leads to demethylations via other catalytic cycles or downstream pathway involving thymine-DNA-glycosylase, which catalyzed base excision with DNA base excision repair [101,120].

**Figure 6:**
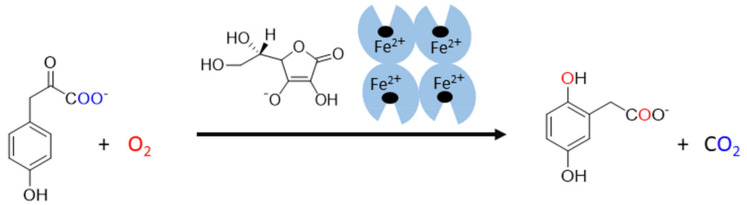
The reaction catalyzed by human 4-hydroxyphenylpyruvate dioxygenase. 4-hydroxyphenylpyruvate is oxidized by molecular oxygen by the enzyme in the presence of ascorbate. The products of this reaction are homogentisate (2,5-dihydroxyphenylacetate) and carbon dioxide. The origin of oxygen is highlighted in a blue and red color.

The last known vitamin C-dependent dioxygenase is **4**-hydroxyphenylpyruvate dioxygenase. It is classified with 2OGO into α-keto acid-dependent oxygenases. It has a ferrous ion again in the active site and needs oxygen. It catalyzes an uncommon reaction in humans, which involves decarboxylation, substituent migration, and aromatic oxygenation in a single catalytic cycle. The 4-hydroxyphenylpyruvate is converted to homogentisate (2,5-dihydroxyphenylacetate, Figure 6) as a part of the tyrosine elimination pathway. Furthermore, here, the superiority of vitamin C over other reducing agents was shown [4,144].

### 3.8. Vitamin C-Dependent Monooxygenases

**Figure 7:**
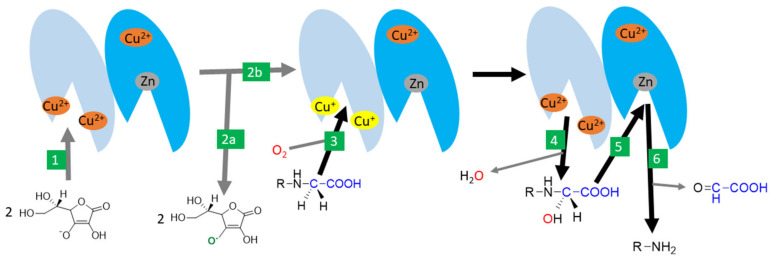
Amidation catalysed by peptidyl-glycine α-amidating monooxygenase (PAM) -the likely mechanism. 1: Ascorbic acid reduces cupric ions in the active site of the peptidyl-glycine α-hydroxylating monooxygenase domain (shown in light blue). 2: two molecules of ascorbic acid are oxidized to ascorbyl radical (2a) and the active enzyme with reduced cuprous ions in the active site is formed (2b). 3: This active site binds the substrate and needs oxygen for the reaction as well. 4: One oxygen is incorporated in the water while the second is incorporated in the substrate. 5: The reaction continues, with the binding of the hydroxylated site to the active center with a zinc atom of the α-hydroxyglycine α-amidating lyase domain of the enzyme (shown in dark blue). 6: This results in the production of the α-amidated enzyme and the release of glyoxylate.

There are two monooxygenases dependent on vitamin C: dopamine-β-hydroxylase and peptidyl-glycine α-amidating monooxygenase (PAM). Both are, hence, associated with the synthesis of hormones. There is a significant homology both in the amino acid sequence and the final structure between them [72]. Dopamine-β-hydroxylase is located in catecholamine storage vesicles of nervous tissue including in chromaffin cells of the adrenal medulla. It is a dimer or tetramer containing identical subunits with each possessing two catalytical copper ions. Ascorbic acid seems to be involved in the reduction of copper. The process appears to be very similar to PAM [4,72,145,146]. PAM is a bifunctional enzyme catalyzing two-step carboxyterminal amidation of peptides. It is expressed in at least seven protein forms, which are the results of different RNA splicing ranging from trans-Golgi membrane-bound to free forms. It is the only known human enzyme able to catalyze α-amidation of peptides [72,147,148,149]. The reaction is crucial for the production of a number of hormones/neurotransmitters (e.g., calcitonin, oxytocin, vasopressin, GLP-1, substance P, neuropeptide Y), and the enzyme is highly expressed in many neurons and endocrine tissues [4,72,150,151,152,153]. The enzyme can also amidate non-proteins such as fatty acid glycines [72]. It is composed of two active enzymatic sites with one catalyzing hydroxylation of the α-carbon of glycine (called peptidyl-glycine α-hydroxylating monooxygenase), and the other dealkylation of the hydroxyglycine intermediate by the release of glyoxylate (α-hydroxyglycine α-amidating lyase or peptidylamidoglycolate lyase, Figure 7). The former active site is composed of two copper ions and the corresponding reaction is ascorbate-dependent. Ascorbate likely enables conversion between a cupric and cuprous state and is reduced stoichiometrically to an ascorbyl radical. Other reductants can replace ascorbic acid but are less potent [4,72,147,148,149].

### 3.9. Vitamin C as an Anti/Prooxidant

**Figure 8:**
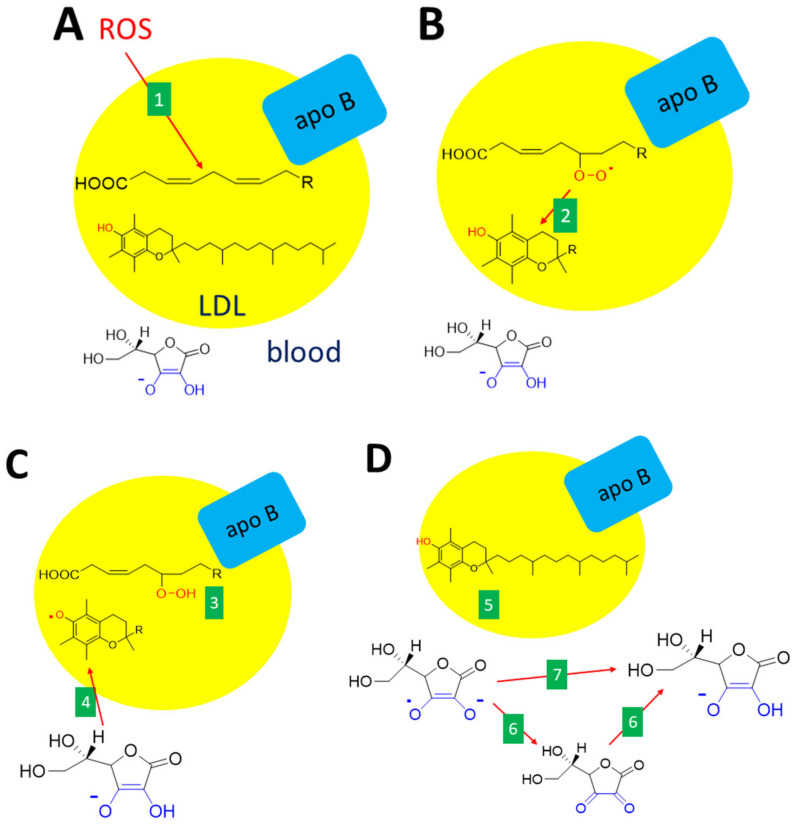
Interconnection between antioxidant effects of vitamin C and E. (**A**): Unsaturated fatty acid within the LDL (low-density lipoproteins) particle is oxidized (e.g., by other ROS, **1**). (**B**): The lipid peroxy radical formed is neutralized by vitamin E (α-tocopherol, **2**). (**C**): The reaction results in a lipid hydroperoxide (**3**) and the formation of the α-tocopheryl radical, which immediately reacts with vitamin C (ascorbate, **4**). (**D**): This leads to the recovery of α-tocopherol (**5**) and the formation of an ascorbate free radical. Ascorbate can be recovered either via dehydroascorbic acid (**6**) or directly (**7**). The are several ways how these reactions can be accomplished either non-enzymatically or enzymatically.

**Figure 9:**
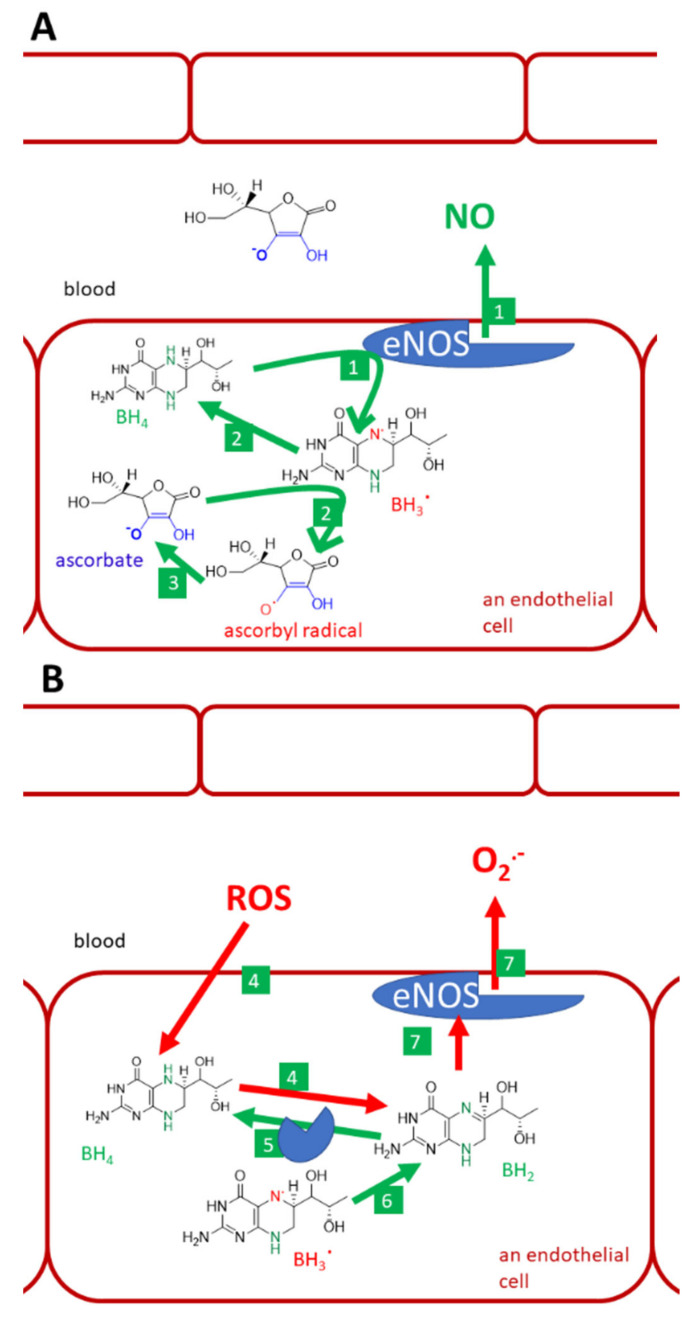

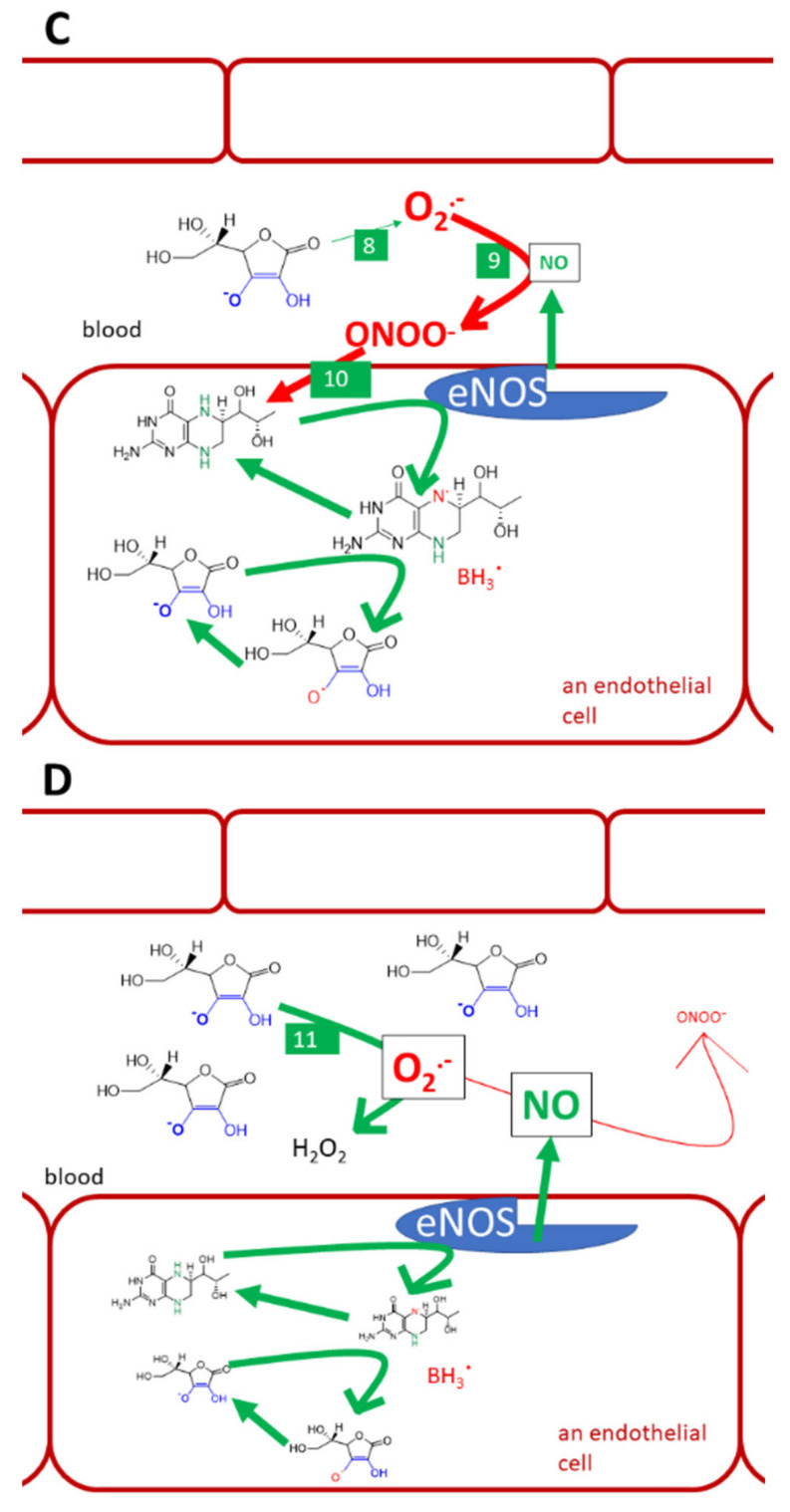
Vitamin C, tetrahydrobiopterin, and endothelial NO-synthase (eNOS). (**A**): normal conditions, (**B**): lack of vitamin C and oxidative stress, (**C**): physiological levels of vitamin C and oxidative stress in the vascular system, (**D**): i.v. administration of high vitamin C doses. Under normal conditions, tetrahydrobiopterin (BH_4_) is used by the eNOS for the synthesis of NO (**1**). Trihydrobiopterin radical (BH_3_**^.^**) can be generated by eNOS. It can be recovered to BH_4_ by both eNOS itself or ascorbate (**2**). Ascorbate is recovered from the ascorbyl radical (**3**) by several pathways discussed in this article. Under the lack of vitamin C and under oxidative stress, BH_4_ is oxidized by reactive oxygen species (ROS). This decreases the availability of this cofactor and may lead to the formation of dihydrobiopterin (BH_2_, **4**). BH_2_ can be reduced back to BH_4_ by dihydrofolate reductase (**5**). Similarly, if BH_3_**^.^** cannot be recovered to BH_4_, it can be oxidized to dihydrobiopterin (BH_2_, **6**). BH_2_ binds to eNOS, but causes uncoupling. Such an enzyme can no longer produce NO, but produces superoxide instead (**7**). Oxidative stress is demonstrated since elevated levels of superoxide in circulation cannot be normalized by physiological concentrations of vitamin C, which has a lower affinity to superoxide (**8**) than superoxide has to NO. As a result, the protective NO is reacting with superoxide into highly reactive peroxynitrite (**9**). Peroxynitrite can also oxidize BH_4_ (**10**) and cause eNOS uncoupling, as was shown in part B (No. 4). However, when vitamin C is given in high doses intravenously, it reaches mM levels and is considered to compete with NO for the superoxide (**11**). The superoxide is neutralized by such a high concentration of vitamin C and NO can exert its endothelial protective function.

The involvement of vitamin C as an endogenous antioxidant, e.g., scavenger of ROS, is still a matter of debate. Its role has been clearly and repeatedly demonstrated in vitro, but in vivo, the situation is less clear [5,6,154,155,156]. A good example is a fetus without an SVCT2 transporter. These fetuses have no vitamin C in the cortex and lungs together with lower levels of vitamin C in the placenta. The markers of lipid peroxidation are clearly elevated in the cortex and placenta but not in the lungs [69]. The effect of ascorbic acid on the recovery of α-tocopherol (Figure 8) and recovery/sparing of tetrahydrobiopterine (Figure 9) is mostly discussed in the literature to have biological relevance [157,158,159] as these activities are associated with protection against endothelial dysfunction.

Oxidative modification of protein moiety of low-density lipoproteins (LDL) can happen in vivo due to oxidative stress via the ROS formed, e.g., by leukocytes. The first line antioxidant in this case is α-tocopherol, which is the most abundant antioxidant in LDL. However, the formed α-tocopherol radical can act as a prooxidant if not scavenged. Here, the ascorbic acid seems to play a crucial role since it recovers α-tocopherol [157]. The formed ascorbate (ascorbyl) free radical, also known as monodehydroascorbate or semidehydroascorbate, is quite stable and can be detected in biological fluids in a concentration of 10 nM [73,154]. This radical is not very reactive and its preferred reaction is the formation of dehydroascorbic acid from two molecules of this radical. The radical can also be directly transformed to ascorbic acid by NADH-dependent or NADPH-dependent enzymes, such as thioredoxin reductase and cytochrome *b_5_* reductase. Again here, redundant pathways apparently exist. In addition, intracellular cytochrome *b*_561_ and/or some not fully defined plasma membrane redox system apparently participate in this process [1,68,73,76].

Recovery and/or protection of tetrahydrobiopterin (BH_4_) by vitamin C is another issue (Figure 9). There are verifications that higher intake/plasma levels of vitamin C are associated with higher levels of tetrahydrobiopterin [158]. The mechanism of how vitamin C maintains BH_4_ levels is not fully clear. Vitamin C recovers BH_4_ from the trihydrobiopterin radical. Although this reaction is relatively specific and thiols like glutathione are not active, the same reduction to BH_4_ can also be performed by the enzyme endothelial NO-synthase (eNOS) itself. Hence, the protection of BH_4_ due to scavenging of other radicals that can oxidize BH_4,_ is a more likely mechanism. For example, the affinity of BH_4_ and ascorbate for superoxide are about the same magnitude. Without BH_4_, which stabilizes all human NOS, eNOS will become uncoupled and produce superoxide instead of NO. ROS can oxidize BH_4_ and, hence, decrease its availability for eNOS. One of the oxidation products is also dihydrobiopterin (BH_2_), which binds to eNOS, but causes the mentioned uncoupling [68,157,158,160,161,162]. However, ascorbate cannot recover BH_4_ from BH_2_ [162]. 

The role of vitamin C in the prevention of endothelial dysfunction is further supported by other findings: (1) it prevents leucocytes adhesion to endothelial cells caused by both oxidized LDL and cigarette smoke [157], (2) it decreases ROS levels in endothelial cells in vitro [163], (3) it recovers flow-dependent vasodilation impaired by smoking and normalizes TBARS (thiobarbituric acid reactive substances, a non-selective marker of lipid peroxidation) in smokers [63], and, (4) similarly, flow-dependent vasodilation was also improved in patients with coronary artery disease after administration of vitamin C [164]. In addition, the phenomenon of nitrate tolerance, which can also be associated with ROS, can be abolished by vitamin C [165]. 

However, there are also reports opposing the antioxidant theory. Multiple studies showed no effect of vitamin C on markers of lipid peroxidation in healthy humans or animals [63,66,166]. Furthermore, very high-mortality of mice injected by *Klebsiella pneumoniae* was markedly abolished by vitamin C, but the effect was not associated with antioxidant activity since vitamin C did not impact neither lipid nor protein oxidation [155]. Notwithstanding the positive effect on flow-mediated dilation, markers of lipid and protein oxidation were not positively modified after the administration of vitamin C in patients with coronary artery disease [164]. The scavenging effects of ascorbic acid on a superoxide are also debatable. In simple experiments, vitamin C is a very active superoxide scavenger. However, in competition with NO, a concentration of vitamin C about 10 mM is needed to block the interaction of NO with the superoxide by effective scavenging of the latter [167]. The reaction of the superoxide with NO is preferred and leads to the production of reactive peroxynitrite [167]. A high concentration of vitamin C can be, however, present intracellularly, as mentioned, in neurons or in plasma after i.v. treatment with high doses of vitamin C [157,158]. On the other hand, glutathione has about 100x lower affinity to superoxide than vitamin C. In general, it appears that ascorbate is the first line hydrophilic antioxidant. Contrarily, glutathione is about twice more active in scavenging peroxynitrite than ascorbate [68].

Higher doses of vitamin C can behave as a prooxidant [156,168,169] and this property is potentially useful for cancer treatment and will be discussed in the chapter entitled *Cancer*.

### 3.10. Vitamin C and Iron Absorption

The last known function of vitamin C is associated again with its potential to reduce ferric ions into ferrous ones. In this way, vitamin C increases iron absorption even with a low amount of vitamin C corresponding to its content in a normal diet [5,154]. 

### 3.11. Vitamin C Deficiency

Vitamin C deficiency is known as scurvy. Typical symptoms of scurvy are muscle weakness, swollen and bleeding gums, loss of teeth, petechial hemorrhaging, spontaneous ecchymoses, anemia, impaired would healing, hyperkeratosis, weakness, myalgia, arthralgia, and weight loss (there can also be a paradoxical weight increase due to swelling) while the early manifestations encompass lethargy, lassitude, and irritability. Dyspnea can be observed as well. The scurvy is potentially fatal and sudden death occurred as a consequence of a cerebral/myocardial hemorrhage or pneumonia. Biochemically, vitamin C plasma levels below 11 µM are considered to coincide with clinical symptoms of scurvy. Similarly, symptoms are not seen unless the total vitamin C content in the body falls below 300–400 mg [2,3,6,157,170].

The direct links of vitamin C with symptoms of scurvy are not easily ascertained due to the complexity of vitamin C functions and its partial replaceability with different reductants [4]. The first mentioned symptoms of vitamin C deficiency such as lassitude and tiredness, occurring at plasma levels approximately below 20 µM, can be due to decreased synthesis of carnitine since a deficiency in carnitine leads to decreased oxidation of fatty acids in muscles and other tissues. It can also be related to decreased synthesis of hormones, e.g., norepinephrine and epinephrine [4,5,6,154]. Peripheral neuropathy after vitamin C encompassing numbness of calves and pain might be related to the epigenetic effect of vitamin C. It is known that vitamin C is needed for myelin formation by Schwann cells and it is thought that these symptoms of peripheral neuropathy can be due to the lack of demethylating effects of vitamin C, which can affect the epigenomic way of activation, proliferation, and differentiation of Schwann cells [102]. 

In the absence of vitamin C, prolyl-4-hydroxylase and lysyl hydroxylases cannot catalyze the hydroxylation. Collagen synthesis is defective and this leads to symptoms of scurvy. Hydroxylation of prolyl residues is needed for the formation of a stable triple-helical procollagen, while lysyl hydroxylation seems to participate in collagen crosslinking as well as in enabling other posttranslational modifications, such as glycosylation and phosphorylation [104,128,129]. Some mild decreases in the hydroxylation of amino acids in collagen were observed in models of vitamin C deficiency. The effect of the absence of vitamin C might be more complex and, in addition to low hydroxylation, there are also reports of decreased synthesis of collagen and other extracellular matrix proteins. Additionally, other connective tissue proteins are physiologically hydroxylated in proline residues, e.g., elastins [4,71,171]. Fetuses without vitamin C in the brain have severe hemorrhages and there is apparently less collagen type IV in the basement membrane of brain vessels [69,70]. Hence, the overt symptoms of vitamin C deficiency such as bleeding or poor healing are associated with abnormalities in connective tissue synthesis.

Although the time when scurvy was a relatively frequent phenomenon has gone, in the relatively close past and even in the current time, the vitamin C deficiency is not fully overcome [3]. It can be observed in refugees and, quite surprisingly, a national survey in the United States in the years 2003–2004 reported that 7% of persons had a vitamin C plasma level below 11.4 µM [61]. The situation seems to be gradually improving since, apparently, vitamin C supplements and socioeconomic status are important factors [61]. As already discussed, smokers are at much higher risk [2]. Additionally, there are several pathologies and other situations in which the level of vitamin C drops in plasma [159]. These are likely associated with an enhanced need for vitamin C. Not surprisingly, surgery, trauma, sepsis, and burns are causing a decrease in blood vitamin C and, in very serious injuries, the drop can be very pronounced [172,173]. Furthermore, acute myocardial infarction is associated with rapid loss of vitamin C both in plasma and tissues [60,174]. Lower levels are also observed in cancer patients. Additionally, 30% of solid cancer patients had vitamin C levels below 11 µM, while 19% of patients with similar values were observed in a group of hematological malignancies [59,175]. Moreover, the other 42% of patients with solid cancer had levels in the range of 11–23 µM and the average level of plasma vitamin C in haematologic cancer patients was 20.5 µM [59,175]. The low intake of vitamin C is markedly contributing to those patients [175]. In patients suffering from atopic dermatitis, the plasma vitamin C levels are normal, but their dermis contains approximately four times less vitamin C [176].

### 3.12. Possible Use of Vitamin C in Therapeutics

#### Cancer

As mentioned, patients with cancer often have lower plasma levels of vitamin C than healthy adults. Moreover, vitamin C deficiency is associated with higher C-reactive protein levels and with higher mortality [2,175,177]. Some clinical trials with high doses of vitamin C failed to show a benefit in cancer patients because vitamin C was administered orally in these studies [154,178,179]. This seems to be largely based on the limits of oral vitamin C to elevate plasma levels. For vitamin C to have a therapeutic impact on cancer, it should be given intravenously to achieve plasma levels in mM. Low concentrations of vitamin C achieved by oral administration are antioxidant while higher procurable by intravenous administration of grams doses are prooxidant and also increase the effect of some cytostatics (e.g., arsenic trioxide, carboplatin, and paclitaxel) and radiotherapy [103]. It was shown that vitamin C given intravenously increases the production of hydrogen peroxide dose-dependently in extracellular fluid but not in blood. High doses of vitamin C leading to its maximal average concentration of 10 mM in plasma resulted up a concentration of about 20 µM of hydrogen peroxide. This was considered a consequence of increased levels of ascorbyl radical, which achieved 250 nM in extracellular fluid [67,180]. The levels of ascorbyl radical and hydrogen peroxide in the tumour or s.c. tissues in mice were even higher [181]. The elevated levels of hydrogen peroxide seem to be one of the possible reasons for the anticancer effect of high doses of vitamin C. In addition to this prooxidant effect, HIF1α and epigenetic pathways can contribute to the possible anticancer effect of vitamin C [177]. The viability of healthy cells is not affected by vitamin C even at 20 mM concentration, i.e., at common concentrations achieved by administration of high doses of vitamin C. Tumor cells are much more, although variably, sensitive to vitamin C, with EC_50_ of vitamin C below 20 mM in all tested cases of human and mouse cancer cells lines [180,181]. A significant number of clinical trials have indicated that vitamin C administered intravenously in high-doses (in most trials, 50–100 g twice or thrice weekly) as a treatment for various cancers including glioblastoma, ovarian, prostate, lung, or rectum cancer is well tolerated with minimal toxicity, improves the quality of life for patients, and has a synergistic therapeutic effect when combined with radiation and/or standard chemotherapy and reduced their side effects. However, most of these studies were not designed as large-scale randomized clinical trials and clear verifications of the clinical efficacy of vitamin C are currently rather limited [177,182,183,184]. On the other hand, the therapy with a high dose of vitamin C can also have some relevant risks for the patients with some type of cancer treatment. Vitamin C in high doses (40 mg/kg/day orally) can significantly reduce the activity of bortezomib treatment in vivo in the human multiple myeloma xenograph model [185].

There is some evidence that vitamin C administered orally may be effective to prevent the development of certain types of malignities (e.g., lung cancer, colorectal adenoma, endometrial cancer) [186,187,188]. On the other hand, there is a reasonable discussion of how much these studies were confounded by other factors such as healthier diet, etc. [5].

Summing up, through low toxicity and low financial cost, high-dose intravenous vitamin C may be possibly a beneficial adjuvant for conventional cancer therapy in certain types of tumors. Oral intake of vitamin C in high doses could be effective in cancer prevention. High-quality placebo-controlled clinical trials are, however, crucially needed to verify and specify the effect of vitamin C both in the treatment and prevention of cancer.

### 3.13. Cardiovascular Diseases

Based on the reported positive antioxidant effect of vitamin C in relation to endothelial dysfunction, many studies investigated the possible protective effect of vitamin C on cardiovascular diseases. Higher levels of plasma vitamin C are correlated with a lower risk of coronary artery disease and mortality in terms of cardiovascular diseases. However, this relationship seems to be valid only for inadequate plasma levels. In adequate vitamin C plasma levels, supplementation with vitamin C has a little effect [2,5,189]. Recent umbrella review brings only limited evidence for the effect of vitamin C supplementation on biomarkers of cardiovascular diseases or its risk factors, such as arterial stiffness, blood pressure, endothelial function, glycemic control, and lipid profile. There is only weak evidence that supplemental vitamin C may improve these biomarkers in selected population subgroups (older and/or obese people, patients with lower vitamin C status at baseline, and patients with a higher risk of cardiovascular disease) [190]. Some recent systematic reviews and meta-analyses suggested that vitamin C significantly decreased the incidence of atrial fibrillation, ventilation time, length-of-stay in the intensive care unit, and hospital length-of-stay, but it had no significant effect on in-hospital mortality or incidence of stroke, acute kidney injury, or ventricular arrhythmia in cardiac surgery patients. The data on the effect of vitamin C on clinical outcomes in patients undergoing cardiac surgery is still insufficient to draw firm conclusions [191,192,193,194].

### 3.14. Infections

The role of vitamin C in the protection of infections was nicely summarized in a recent review [157]. It can be shortly summarized that vitamin C is important for the differentiation and function of immune cells and epithelial barrier cells. Patients with infections have lower levels of vitamin C and animal models have largely shown a protective effect of vitamin C on different infections or intoxications with bacterial toxins [155,195]. The human studies are, however, much less clear. The Cochrane library has not documented the preventive effect of vitamin C administration on the incidence of the common cold and found only a very mild effect on the duration of the common cold. The effect of vitamin C in the prevention and treatment of pneumonia is uncertain as well [196,197]. Notwithstanding that the effect is absent in the general population, vitamin C can be effective under specific conditions, such as low levels of vitamin C, e.g., in people under physical stress [6,191,195,198]. The recent clinical trial did not find the effect of intravenous vitamin C infusion on organ failure and biological markers of inflammation and vascular injury in patients with sepsis and acute respiratory distress syndrome, but vitamin C compared with the placebo was associated with a significant reduction in 28-day all-cause mortality [199]. There are also some other clinical limited reports showing that vitamin C can improve the consequences of sepsis [200]. 

Due to the possible positive effects on respiratory diseases, acute respiratory distress syndrome and sepsis, low cost and excellent safety profile, administrations of vitamin C to patients with hypovitaminosis C, and severe respiratory infections, e.g., COVID-19, could appear warranted. However, there is currently only one small clinical trial reporting the possible effect on mortality in more severely ill COVID-19 patients who received vitamin C intervention. Currently, there are many randomized controlled trials registered globally assessing the effect of intravenous vitamin C in patients with COVID-19 in which outcomes are awaited with interest [201,202].

Vitamin C was also claimed to decrease the pH of urine and, hence, to be useful in the prevention of recurrent urinary infections. However, it was shown that even high doses of vitamin C do not decrease the pH of urine [203].

### 3.15. Other

Vitamin C is frequently added to oral preparations containing iron in order to increase iron absorption [5,154]. In a recent meta-analysis, patients who received intravenous vitamin C perioperatively had significant pain reduction and decreased morphine requirements [204].

### 3.16. Toxicity

A single oral dose of vitamin C of 5–10 g produces transient osmotic diarrhea and/or abdominal bloating with pain but, otherwise, even such a high dose is considered to be safe. However, this is not recommended. Intake with food can decrease these adverse reactions [3,5,156,172]. High, in particular i.v., the dose is described to produce polyuria by the same mechanism [203,205]. Interestingly, even very high doses of i.v. vitamin C (ranging from 1 to 200 g and given repeatedly) are apparently well tolerated in most patients [206]. Vitamin C is metabolized partly into oxalate (Figure 4) in the human organism. Vitamin C increases oxalate levels in urine dose-dependently and there are concerns about possible urinary stone formation. The question is somehow controversial since early methods overestimated oxalate levels in urine due to experimental artifacts [5,55,203,205,207,208]. Interestingly, parenteral preparations of vitamin C might also contain oxalate, likely because oxalate is easily formed in vitro from vitamin C at a higher pH [205]. In any case, although urinary oxalate is the crucial player in calcium stone formation, the risk of urinary stone formation seems to be very low after intake, even of high vitamin C doses. The major reason is that, in general, long-term high concentrations of oxalate in urine are needed for developing the stones. It cannot be, however, ignored since some persons might be at higher risk. For example, higher basal urinary levels of oxalate are found in “urinary stone formers” and, similarly, vitamin C administration is inducing higher urinary levels of oxalate in these patients than in non-formers [203,206,207,208]. A prospective cohort study suggested that intake of oral doses of vitamin C higher than 1 g increases the risk of stone formation significantly by 41% [209]. For this reason, higher doses of vitamin C than 1 g daily should not be routinely recommended [5]. Of note, high doses of vitamin C were also described to increase urate excretion transiently [5,55].

Intravenous vitamin C or very high oral vitamin C doses can precipitate hemolysis in glucose-6-phosphate deficiency patients [5]. Oral ascorbic acid can worsen hemolysis in patients suffering from paroxysmal nocturnal hemoglobinuria [210]. In vitro, the effect of vitamin C on the lysis of red blood cells from these patients is concentration-dependent with worsening in low concentrations while inhibition occurred in high concentrations [105,210].

It should also be mentioned that a high intake of vitamin C in mothers can result in rebound scurvy in new-borns [3].

### 3.17. Determination of Vitamin C in Biological Material

In general, determining the presence of L-ascorbic acid in a biological sample is a difficult task due to the possible interference with several variables. The stability of L-ascorbic acid is the major problem. Since vitamin C is a known antioxidant, its oxidation in the human body into dehydroascorbic acid has been proposed as an in vitro marker of oxidative stress. However, the oxidation occurs rather quickly and, hence, also artificially after collecting the sample. Although dehydroascorbic acid is far more stable than L-ascorbic acid, it may still undergo irreversible hydrolysis in 2,3-diketogluconate (Figure 4). Dehydroascorbic acid concentration in healthy adults is normally below 2% of that of L-ascorbic acid [211]. Moreover, the simultaneous determination of both is difficult because of the different physicochemical properties of these analytes. Therefore, the simultaneous determination is achieved via a double analysis of one sample with a redox reaction conversion (oxidation of the former/reduction of the latter) and subsequent subtraction. However, this procedure often lacks specificity and is prone to the interference of other reducing agents. This problem can be solved by combining different detection techniques. It should be pointed out, though, that vitamin C in urine can interfere with urine stripe tests. For example, vitamin C can cause false positive or false negative results during the detection of glucose, leukocytes, nitrite, and bilirubin [212].

The stability of L-ascorbic acid in aqueous solutions can be affected by a number of factors including light, temperature, pH, and presence of oxygen and metal ions, which must be considered during its determination. In this regard, the concentration of L-ascorbic acid can decrease during exposure to UV light (decrease to 80%), natural light in a transparent container (decrease to 84%), and in a brown flask (decrease to 96%). Most studies concerning biological material used samples cooled to 4 °C immediately after collection because the concentration of L-ascrobic acid decreases rapidly at higher temperatures. These samples can be stable only for 1 h at room temperature. Most of the published protocols for the extraction of L-ascrorbic acid from biological material use acidic pH 2.1 to improve the stability of L-ascorbic acid. On the other hand, both L-ascorbic and dehydroascorbic acid display better stability at higher concentrations. Their stability decreases significantly when their concentration is lower than 0.1 mg/L. L-ascorbic acid can also be degraded in the presence of oxidizing enzymes and metal ions, especially Cu^2+^ and Fe^2+^ [213,214]. The usage of anticoagulants for blood collection also plays an important role. Heparin and ethylenediaminetetraacetic acid (EDTA) on ice are the most suitable since L-ascorbic acid is unstable with gel or fluoride anticoagulants [215]. Thus, a number of preventive steps should be followed to avoid the degradation of the analytes when processing biological material for determining L-ascorbic and dehydroascorbic acid. In general, the rapid transport of the sample to the laboratory in a dark container at low temperature should be procured. Some stabilizers, such as meta-phosphoric acid trichloroacetic acid, homocysteine, trifluoroacetic acid, oxalic acid, and EDTA (for chelating undesirable metal ions), are often added prior to sample storage or preparation. Sometimes these substances can also be combined with buffers or organic solvents such as methanol and acetonitrile [213,214,215]. The stability of L-ascorbic acid and dehydroascorbic acid in biological samples was studied in detail by Pullar et al. (2018) and Bernasconi et al. (2018) [216,217]. These authors pointed out, for example, the necessity of immediate separation of stabilized plasma from blood cells, the influence of hemolysis on ascorbate oxidation due to the release of catalytic iron from haemoglobin, and necessity to keep EDTA anticoagulant samples cold during handling.

Numerous methods for the determination of L-ascorbic acid in foods and pharmaceuticals have been published [218,219,220,221]. However, the determination of vitamin C in biological samples has not garnered further attention, likely because of its complexity. The most common biological materials used for its determination are serum, plasma, urine, red and white blood cells, breast milk, and sweat [222,223]. The recent methods include capillary electrophoresis, liquid chromatography, and electrochemical biosensors in addition to commercially available kits that can be used in routine applications. Some selected methods used for vitamin C determination in human biofluids are presented in Table 3.

Due to its sensitivity and good selectivity, capillary electrophoresis (CE) coupled with electrochemical detection (ECD) has been one of the most widely used techniques in the determination of AA in biological samples. However, the high separation voltage could interfere with the electrochemical detection and the analysis of biomatrix samples could contaminate the electrode surface. These methods are only used in the determination of L-ascorbic acid because dehydroascorbic acid is electrochemically inactive [224].

The determination of vitamin C by high-performance liquid chromatography HPLC (high-performance liquid chromatography) was summarized in a review by Nováková et al., 2008, where the general aspects of chromatographic determination are summarized in detail [256].

UV (ultraviolet) and ECD (electrochemical detection) are the most common detection systems used in HPLC. Unfortunately, none of them enables the simultaneous detection of L-ascorbic and dehydroascorbic acid in a single run. The absorbance maximum of L-ascorbic acid is in the range of 244–265 nm as a function of pH. UV detection of dehydroascorbic acid requires its prior derivatization due to its poor absorption at a wavelength of 185 nm. Unlike L-ascorbic acid, dehydroascorbic acid is electrochemically inactive and ECD detection is also not possible [256]. Fluorescent detection is not widely used because it requires an additional time-consuming derivatization step. The specific reaction of dehydroascorbic acid with dimethyl-o-phenylenediamine results in a fluorescent quinoxaline derivative that is quantified by HPLC [257]. Mass spectrometry (MS) detection has been rarely used, even though it enables the simultaneous detection of both substances. Only a few methods for the determination of DHA and AA in human fluids with MS detection were presented in the medical literature [214,231]. The difficulty of LC-MS method development is related to the matrix effect induced signal suppression, but other factors, such as non-standard ionization patterns, resulting in complex ion species and in-source interconversion play an important role. The MS detection is generally carried out in a negative mode with electrospray ionization [213,214]. Some MS detection methods use the subtraction approach in a similar manner to UV detection [231]. Typically, chromatographic methods carry out the separations in a reverse phase mode in which L-ascorbic and dehydroascorbic acids are poorly retained due to a very hydrophilic nature. A very low pH is needed to avoid degradation of AA in samples and to allow some retention. On the other hand, ion exchange, ion pair, and ion exclusion chromatography more suitable for retention of these polar species require either a complex mobile phase composition, including ion-pairing reagents, EDTA, or the presence of inorganic buffers, such as phosphate or borate buffers. All these reagents are incompatible with MS detection. On the other hand, hydrophilic interaction liquid chromatography (HILIC) would be very convenient to determine L-ascorbic acid. However, the bioanalytical results are rather scarce [225].

Extraction techniques for L-ascorbic and dehydroascorbic acids present in biomatrices applied prior to HPLC and CE (capillary electrophoresis) analysis are simple and mostly comprise dilution, protein precipitation, centrifugation, and filtration. The sample preparation can also be performed using solid phase extraction [237]. Since the target analytes can degrade during the sample preparation process, stabilizers and/or chelators need to be added to the sample [223,226,228].

An internal standard is not routinely used to determine L-ascorbic acid in biological material because it is difficult to find a compound that is suitable for both extraction and analysis steps similar to L-ascorbic acid. For example, its stereoisomer iso-ascorbic acid (Figure 1B), has been used in bioanalysis. However, it can be naturally present in some biological samples. Other substances, such as tris(2-carboxyethyl)phosphine hydrochloride, have also been used, even though they can undergo similar redox reactions as L-ascorbic acid. Thus, internal standard quantification is difficult [213,225]. This problem can be solved by using stable isotopically-labelled internal standards during MS detection.

Electrochemical sensors are a vast area for the exploration of vitamin C determination. Unfortunately, they were used for biological samples only to demonstrate the possibility of their use but not their application in routine practice. Nevertheless, they possess many advantages such as simplicity, cost-effectiveness, high sensitivity, easy miniaturization, reliability, and reproducibility. Moreover, the recent innovations in electrode nanomaterials improved both sensitivity and selectivity [239] and yielded one of the most sensitive techniques for L-ascorbic acid detection in biological materials. As a downside, the use of these sensors in routine practice or in large clinical studies has yet to be tested. In this regard, a cost-effective preparation of carbon quantum dots to determine Fe^3+^ ions and L-ascorbic acid detection in living cells showed a limit of detection of 196 nmol/L [258]. 

Finally, commercially available vitamin C kits are often used in routine and research clinical applications. 

These commercial kits can be classified as follows.

1. HPLC-UV kits: These kits contain the chromatographic column, the mobile phase, the extraction reagents, and the control material. They are usually based on a reverse-phase mode and have higher selectivity and specificity than other types of L-ascorbic acid kits. However, they need complex instrumentation and are very expensive.
2. Colorimetric kits: They enable the detection of L-ascorbic acid in a wide range of biological fluids, tissues, and cells. They are available in 96-well or dipstick formats [250]. The determination is based on the chelation of ferrous iron with a colorimetric probe to produce a compound exhibiting a strong absorbance. The manufacturer of FRASC L-ascorbic acid Assay kit II claims a detection threshold of 0.2–20 nmol.
3. ELISA kits: A classical ELISA-type immunokit is based on antigen-antibody interaction. The competitive ELISA kits utilize a polyclonal anti-vitamin C antibody and a vitamin C-HRP conjugate. Their main disadvantage is the cost and the possible interaction with interferences that reduce the selectivity.

## 4. Conclusions

Vitamin C has become a very popular over-the-counter preparation mainly due to its claimed strengthening effects on immunity with preventive activity toward different infections, and possible effects on other diseases encompassing cardiovascular diseases and cancer. The latter are traditionally linked to its antioxidant properties, which have been repeatedly confirmed in vitro. It should be, however, mentioned, that vitamin C can also behave as a prooxidant. This effect is apparent in high doses and seems to be the base for its possible use as an anti-cancer drug when given intravenously. Although there are also claims of its protective cardiovascular effects, definite proofs are missing. The protective effects of vitamin C against different infections are generally low, but might be tangible in some specific conditions. Vitamin C is an essential or optimal cofactor for many enzymes involved in several processes, including the correct formation of connective tissue and hormone synthesis. Recently, its role in several epigenetic processes was revealed. Future studies will likely specify the role of vitamin C in more depth in these processes. In conclusion, vitamin C is largely non-toxic, but its high doses should be avoided due to the rare but well-documented formation of kidney stones and rebound scurvy of new-borns.

## Figures and Tables

**Table 1:** Vitamin C content in selected fruits, vegetables, and medicinal plants.

Latin Name	Family	Vernacular Name	Vitamin C Content	References
Fruits				
*Terminalia ferdinandiana* Exell	Combretaceae	Kakadu plum	1360–22,490 ^b^	[17,41]
*Myrciaria dubia* (Kunth) McVaugh	Myrtaceae	Camu-camu	850–5000 ^a^	[18,42]
*Malpigia emarginata* DC.	Malpighiaceae	Acerola	820–4023 ^a^	[19,34]
*Averrhoa bilimbi* L.	Oxalidaceae	Bilimbi	2698 ^c^	[22]
*Averrhoa carambola* L.	Oxalidaceae	Star fruit	1626 ^c^	[22]
*Psidium guajava* L.	Myrtaceae	Guava	89–980 ^a^	[18,23,43]
*Anacardium occidentale* L.	Anacardiaceae	Cashew apple	555 ^a^	[25]
*Phyllanthus emblica L.*	Phyllanthaceae	Emblic	469 ^a^	[35]
*Ribes nigrum* L.	Grossulariaceae	Black currant	148–310 ^a^	[24]
			60–250 ^d^	[32]
*Actinidia deliciosa* (A.Chey.)C.F.Liang et A.R.Ferguson	Actinidiaceae	Kiwi	60–78 ^a^	[11,25]
*Fragaria virginiana* Duchesne	Rosaceae	Strawberry	65 ^a^	[25]
*Citrus* x *sinensis* (L.)Osbeck.	Rutaceae	Orange	41–58 ^a^	[11,25,44]
*Citrus limon* (L.)Osbeck.	Rutaceae	Lemon	30 ^d^31 ^a^	[44,45]
*Citrus reticulata* Blanco	Rutaceae	Common mandarin	27 ^a^	[25]
*Malus domestica* Borkh.	Rosaceae	Apple	11–35 ^a^	[46]
*Pyrus communis* L.	Rosaceae	Pear	7–29 ^a^	[46]
Vegetables				
*Brassica oleracea* var. *italica* Plenck.	Brassicaceae	Broccoli	25–130 ^a^	[26]
*Brassica oleracea* var. *acephala* (DC.)Alef.	Brassicaceae	Kale	51–120 ^a^	[26]
*Capsicum annuum* L.	Solanaceae	Pepper	107–154 ^a^	[27]
*Solanum tuberosum* L.	Solanaceae	Potato	8–30 ^a^	[11,29]
*Solanum lycopersicum* L.	Solanaceae	Tomato	9–17 ^a^	[47,48]
Fermented vegetable				
*Brassica oleracea* var. *capitata* (L.)Alef.	Brassicaceae	Sauerkraut	103–277 ^b^	[28]
Medicinal plants and herbs				
*Hippophaë rhamnoides* L.	Eleagnaceae	Sea buckthorn	70–1320 ^d^	[20,33]
*Rosa canina* L.	Rosaceae	Rosehip	40–360 ^a^	[21,49]
*Coriandrum sativum* L.	Apiaceae	Coriander	48–98 ^a^	[30,50]
*Allium schoenoprasum* L.	Amaryllidaceae	Chives	93 ^a^	[30]
*Petroselinum crispum* (Mill.)Nym.	Apiaceae	Parsley	59 ^a^	[30]

mg/100 g of ^a^ fresh weight, ^b^ dry weight, ^c^ juice, mg/100 mL of ^d^ juice.

**Table 2:** Overview of the most important groups of iron-dependent and 2-oxoglutarate-dependent dioxygenases.

Physiological Role	Reaction	Enzymes (Subfamily)	References
Collagen stabilization and maturation	Hydroxylation	CP4H, CP3H, PLODs	[104,127,128,129]
Regulation of HIF-1α signaling pathway	Hydroxylation	PHDs, FIH	[101,110,111,130,131,132,133]
Regulation of epigenetic modifications—“epigenetic erasers”	Histone demethylation	JHDMs, KDMs	[102,103,107,108,109,112,113,114,134,135,136,137,138]
	DNA and RNA demethylation	AlkBHs, FTO	[101,103,121,122,123,124,125,126]
	Ribosomal hydroxylation	MINA53, NO66	[115,116,139,140]
	Cytosine demethylation	TETs	[101,102,103,114,117,118,119,120]
Carnitine synthesis	Hydroxylation	TMLHE, BBOX	[4,141,142,143]

CP4H, CP3H—collagen prolyl-4-hydroxylase and -3-hydroxylase. PLODs, pro-collagen lysine 2-oxoglutarate 5-dioxygenases. HIF, hypoxia-inducible factor. PHDs, prolyl hydroxylase domain-containing proteins. FIH, factor inhibiting HIF. JHDMs, Jumonji-C domain histone demethylase. KDMs, lysine demethylases. AlkBHs, alkylated DNA repair protein AlkB homologs. FTO, fat-mass and obesity-associated protein. MINA53, Myc-induced nuclear antigen 53. NO66, nucleolar protein 66. TET, ten-eleven translocases. TMLHE, trimethyllysine hydroxylase epsilon. BBOX, gamma-butyrobetaine dioxygenase.

**Table 3:** Summary of methods for determination of vitamin C in human biological materials.

Technique	Sensitivity (AA in μM if not Specified)	Advantages	Disadvantages	References
LC-UV/PDA	4.95 *	Commonly affordable technique,	AA determination only (poor absorption properties of DHA),	[211,225,226,227]
	4.0 *			
	5.0 **			
	31.81 *			
LC-ECD	9 × 10^−2^ *	selectivity and sensitivity, easy miniaturization	DHA is electroinactive, contamination of electrode by real samples	[226,228,229,230]
	1.34 *			
	2.5 × 10^−2^ *			
	0.50 *			
LC-MS	0.5 **	selectivity, possibility of simultaneous determination of AA and DHA possibility of labeled internal standards usage	Costly device, highly skilled personnel, complicated DHA ionization	[213,214,231]
	DHA: 5 **			
	113 **			
CE-ECD	CZE-ECD:	Small sample and solvent volumes, good sensitivity	High separation voltage could interfere with the detection of an electrochemical signal, contamination of electrode by real samples, DHA is electroinactive	[224,232,233,234]
	1.7 ***			
	0.49 ***			
	0.50 ***			
CE-CL	MCE-CL:	Small sample and solvent volumes, good sensitivity	No natural luminescence of AA (necessity of reaction with luminol—AA enhancing effect), contamination of electrode by real samples	[232,235,236]
	1.3 ***			
	CZE-CL:			
	0.01 ***			
CE-UV	MEKC-UV:	Small sample and solvent volumes	Low sensitivity, poor absorption properties of DHA, AA determination only	[232,237,238]
	5.0 ***			
	0.85 ***			
biosensors	0.12***	Small sample and solvent volumes, low price, portable, good sensitivity, possible to detect AA in vivo	Mostly using ECD—impossible to detect DHA, not commercially available, not tested for large biological sample series	[239,240,241,242,243,244,245,246,247]
	7.4 × 10^−2^ ***			
	8.5 × 10^−4^ ***			
	5.0 × 10^−4^ ***			
	0.02 ***			
	5.68 × 10^−3^ ***			
	9.0 × 10^−3^ ***			
	13.5 × 10^−3^ **			
	0.85 × 10^−3^ ***			
HPLC-UV kits	2.84 *	See LC-UV	See LC-UV, very high cost	[248,249]
	2.27 *			
colorimetric/FLD kits	2.0 × 10^−4^ ***	One kit usable for different matrices (fluids, cells, tissues), commonly available technique, small sample, and solvent volumes, low operation cost	Impossible to differentiate AA and DHA, suitable for large sample series—expiration of the kit after opening	[250,251,252,253]
	5.0 *** (FLD)			
	3.2 ***			
immunoassays kits	0.57 ***	One kit usable for different matrices (fluids, cells, tissues), commonly available technique, small sample, and solvent volumes, low operation cost	Cross-reactions, impossible to differentiate AA and DHA, suitable for large sample series—short expiration of the kit after opening	[254,255]
	1.08 ***			

* LOQ (limit of quantification), ** LLOQ (lower limit of quantification), *** LOD (limit of detection). AA, ascorbic acid. DHA, dehydroascorbic acid. LC-UV/PDA, liquid chromatography with photodiode array/ultraviolet detection. FLD, fluorescence detection. LC-ECD, liquid chromatography with electrochemical detection. LC-CL, liquid chromatography with chemiluminescence detection. LC-MS, liquid chromatography with mass spectrometry detection. CE-ECD, capillary electrophoresis with electrochemical detection. CE-CL, capillary electrophoresis with chemiluminescence detection. CE-UV, capillary electrophoresis with ultraviolet detection. CZE-ECD, capillary zone electrophoresis with electrochemical detection. MCE-CL, microchip capillary electrophoresis with chemiluminescence detection. CZE-CL, capillary zone electrophoresis with chemiluminescence detection. MEKC-UV, micellar electrokinetic chromatography with ultraviolet detection. ECD, electrochemical detection. LC-UV, liquid chromatography with ultraviolet detection. HPLC-UV, high-performance liquid chromatography with ultraviolet detection.
